# Validation and recalibration of OxMIV in predicting violent behaviour in patients with schizophrenia spectrum disorders

**DOI:** 10.1038/s41598-021-04266-9

**Published:** 2022-01-10

**Authors:** Jelle Lamsma, Rongqin Yu, Seena Fazel, Genetic Risk and Outcome of Psychosis (GROUP) investigators, Therese van Amelsvoort, Agna Bartels-Velthuis, Wiepke Cahn, Lieuwe de Haan, Frederike Schirmbeck, Claudia Simons

**Affiliations:** 1 Department of Criminology and Sociology, Middlesex University, London, UK; 2 Department of Psychiatry, University Medical Centre Utrecht, Utrecht, The Netherlands; 3 Department of Psychiatry, Warneford Hospital, University of Oxford, Oxford, UK; 4 Department of Psychiatry and Neuropsychology, Maastricht University Medical Centre, Maastricht, The Netherlands; 5 University Centre for Psychiatry, University of Groningen, Groningen, The Netherlands; 6 Department of Psychiatry, Academic Medical Centre, Amsterdam, The Netherlands; 7 Arkin Institute for Mental Health, Amsterdam, The Netherlands; 8 GGzE Institute for Mental Health Care, Eindhoven, The Netherlands

**Keywords:** Psychology, Human behaviour

## Abstract

Oxford Mental Illness and Violence (OxMIV) addresses the need in mental health services for a scalable, transparent and valid tool to predict violent behaviour in patients with severe mental illness. However, external validations are lacking. Therefore, we have used a Dutch sample of general psychiatric patients with schizophrenia spectrum disorders (*N* = 637) to evaluate the performance of OxMIV in predicting interpersonal violence over 3 years. The predictors and outcome were measured with standardized instruments and multiple sources of information. Patients were mostly male (*n* = 493, 77%) and, on average, 27 (SD = 7) years old. The outcome rate was 9% (*n* = 59). Discrimination, as measured by the area under the curve, was moderate at 0.67 (95% confidence interval 0.61–0.73). Calibration-in-the-large was adequate, with a ratio between predicted and observed events of 1.2 and a Brier score of 0.09. At the individual level, risks were systematically underestimated in the original model, which was remedied by recalibrating the intercept and slope of the model. Probability scores generated by the recalibrated model can be used as an adjunct to clinical decision-making in Dutch mental health services.

## Introduction

Structured tools for violence risk assessment are increasingly used to inform decisions around admission, discharge and treatment of psychiatric patients^1^. However, their suitability for patients with schizophrenia spectrum disorders is questionable. The most widely used tools, such as the Historical, Clinical and Risk Management-20 (HCR-20), Violence Risk Appraisal Guide (VRAG) and Level of Service Inventory (LSI)^2^, were developed in other populations and with methods that are now considered low quality (e.g., absence of a study protocol, small and selected samples, vague or undefined risk categories)^3^. Moreover, they have rarely been validated in patients with schizophrenia spectrum disorders and have low-to-moderate discrimination across psychiatric populations. A systematic review identified two tools, the HCR-20 and VRAG, that were validated in outpatients with schizophrenia spectrum disorders (twice each). The median areas under the curve (AUCs) had wide ranges (interquartile range [IQR] = 0.60–0.77). In outpatients with any diagnosis, the median AUCs of the 10 included tools ranged from 0.62 to 0.85^4^. Another systematic review found an aggregated median AUC of 0.69 (IQR = 0.62–0.75) for 7 tools validated in diagnostically heterogenous samples of inpatients^5^. None of the validation studies, in either review, reported calibration measures. Finally, current tools are resource intensive. They typically take several hours to complete and cost hundreds of dollars in manuals and training^6^. By contrast, other areas of medicine, in particular cardiology, have developed scalable tools that can be used by clinicians to discuss health risks with patients^7^.

A tool that overcomes many of these limitations is Oxford Mental Illness and Violence (OxMIV). It is freely available online (https://oxrisk.com/oxmiv-7/), relies on routinely collected information, and requires no formal training. The model was derived and externally validated in a total population cohort of over 75 000 Swedish individuals with schizophrenia spectrum or bipolar disorder. The candidate variables, statistical analyses and output were specified beforehand. Upon entry of the 16 items, OxMIV estimates the probability of violent offending within 1 year. This estimate is expressed as a percentage, capped at 20%. A classification of ‘low risk’ (< 5%) or ‘increased risk’ (≥ 5%) is also given. In external validation, OxMIV showed excellent discrimination—the AUC was 0.89 (95% confidence interval [CI] 0.85–0.93)—and calibration^8^. A recent study in Germany found moderate discrimination (AUC = 0.72) for the prediction of inpatient violence in a forensic setting^9^.

However, further studies are needed to validate OxMIV for different countries, care settings and forms of violent behaviour. The last are relevant because all violence (not solely incidents leading to arrest and conviction) has negative consequences, including treatment disruption, morbidity in victims, costs to services^10^ and stigmatisation of patients^11^, and may accurately be predicted by OxMIV. Therefore, we have evaluated the performance of OxMIV in predicting interpersonal violence over a 3-year period in a Dutch sample of general psychiatric patients with schizophrenia spectrum disorders. We also explored the feasibility of adjusting OxMIV for this population and outcome.

## Methods

### Setting and participants

Data were collected as part of a larger research project, called Genetic Risk and Outcome of Psychosis (GROUP). The GROUP project was conducted by four university hospitals and affiliated mental healthcare centres (*k* = 36) in the Netherlands. These institutions are located in representative geographical areas of the country and provide access to psychiatric treatment in a variety of settings (e.g., psychiatric hospitals, outpatient clinics, residential care) to approximately 75% of the population. Throughout 2004, consecutive patients were invited to participate if they met the following criteria: (1) age between 16 and 50; (2) good command of the Dutch language; and (3) Diagnostic and Statistical Manual of Mental Disorders, Fourth Edition, Text Revision (DSM-IV-TR)^12^ diagnosis of schizophrenia or other non-affective psychotic disorder. Their parents and siblings were also invited. In total, 1013 patients, 907 parents and 1061 siblings enrolled. Assessments took place at the university hospitals, with a follow-up at 3 years. The protocol for the GROUP project was approved centrally by the ethics committee of the University Medical Centre Utrecht and implemented in accordance with relevant guidelines. All participants gave written informed consent before the first assessment.

### Predictors and outcome

We selected variables whose definitions most closely matched those in the derivation study. The definitions of the predictors (Table S1) and the instruments used to measure them (Table S2) can be found in the supplement. The psychometric properties of the instruments and training of research personnel have been described elsewehere^13^. The patients themselves provided information on all predictors, apart from ‘parental drug or alcohol misuse’ (parents), ‘parental violence’ (parents) and ‘sibling violence’ (siblings). For the predictors ‘parental violence’, ‘sibling violence’ and ‘personal income’, data were only collected at three of the four university hospitals. We excluded cannabis from the predictors ‘previous drug misuse’ and ‘parental alcohol or drug misuse’, as their prevalence would otherwise have been considerably higher than in the derivation sample (46% vs. 12% and 20% vs. 11%, respectively). Furthermore, we have previously shown that, in the current sample, the contribution of cannabis misuse to violence is small and nonsignificant when adjusted for background factors^14^. A possible explanation for both observations is that, unlike in Sweden, cannabis use is not criminalised in the Netherlands.

The outcome was physical abuse of another person (i.e. interpersonal violence) during the 3 years of follow-up, ascertained from clinical case notes and patient interviews. The definition (physical abuse vs. violent offending) and time period (3 years vs. 1 year) thus differed from the outcome OxMIV is designed to predict.

### Statistical analysis

We aimed to validate and, if necessary, update OxMIV for a different population (i.e., general psychiatric patients with schizophrenia spectrum disorders in the Netherlands) and outcome (i.e., interpersonal violence over 3 years). For model updating, we followed an incremental strategy suggested previously^15,16^. This strategy involves up to three steps: (1) recalibrating the intercept; (2) recalibrating the intercept and slope; and (3) re-estimating one or more coefficients. Performance was assessed in terms of calibration, both ‘in the large’ (with the ratio between predicted and observed events across the sample and the Brier score) and at the individual level (through calibration plots), and discrimination. We calculated the following discrimination metrics: AUC, sensitivity, specificity, and positive (PPVs) and negative (NPVs) predictive values. Wilson’s method^17^ was used to construct 95% confidence intervals around the last four of these. Discrimination was also visualised with a receiver operating characteristic (ROC) curve. To aid interpretation, we presented results for the following probability thresholds: 5% (the default), 10%, 15% and 20% (the cap).

Based on its distribution in the Dutch population, the predictor ‘personal income’ was converted into deciles. Patients with ‘unstable’ incomes belonged to deciles 1–3, and those with ‘stable’ incomes to deciles 4–10^18^. The corresponding model coefficients were averaged. Since the predictor ‘recent (substance) dependence treatment’ was not available, we assigned the derivation sample proportion to all patients. The same was done with patients who had ever been admitted to a psychiatric hospital for the predictor ‘currently an inpatient’. Others were assumed to be outpatients. For partially missing predictors, we used multiple imputation by chained equations. As recommended, the outcome was excluded from the imputation models^18^. We averaged values across 20 imputations. Among the predictors measured at all sites, proportions of missing data were modest (≤ 13%) (Table S3). Insofar data were missing due to local practice, they can reasonably be assumed to be missing at random. Missingness on most predictors correlated significantly (*p* < 0.05) with values on at least one other predictor (Table S4).

Outcome data were available for 637 (63%) patients. As outcomes should not be imputed in external validation^19^, these patients formed the sample used in the analyses. They typically had attained a higher level of education (χ^2^ [2] = 12.18, *p* = 0.002) and were less likely to receive benefits (χ^2^ [1] = 5.42, *p* = 0.020) than patients without outcome data. No significant differences were observed for any of the other predictors (Table S5).

Analyses were carried out in SPSS 21.0 and Stata 12.1. We adhered to the TRIPOD statement for validation studies^20^.

## Results

Table 1 outlines summary statistics for the predictors in the patient sample (*N* = 637). Patients were mostly male (*n* = 493, 77%) and, on average, 27 (SD = 7) years old. Previous violence (*n* = 115, 21%) and drug misuse (*n* = 118, 20%) were each present in about one in five patients. Almost all patients (*n* = 565, 95%) had taken antipsychotics in the past 6 months. Fifty-nine (9%) patients physically assaulted another person during the 3 years after baseline.

**Table 1:** Summary statistics for the predictors in the current sample (*N* = 637).

Predictor	Summary
Male sex	493 (77%)
Age	M (SD) = 27 (7)
Previous violence	115 (21%)
Previous drug misuse	118 (20%)
Previous alcohol misuse	79 (13%)
Previous self-harm	142 (23%)
**Educational level**	
Lower secondary	265 (42%)
Upper secondary	177 (28%)
Post-secondary	189 (30%)
Parental drug or alcohol misuse	63 (18%)
Parental violence	13 (5%)
Sibling violence	47 (12%)
Recent antipsychotic treatment	565 (95%)
Recent antidepressant treatment	147 (23%)
Unstable income	369 (80%)
Benefit recipient	276 (46%)

Note: Patient numbers for each row varies due to missing data.

**Figure 1:**
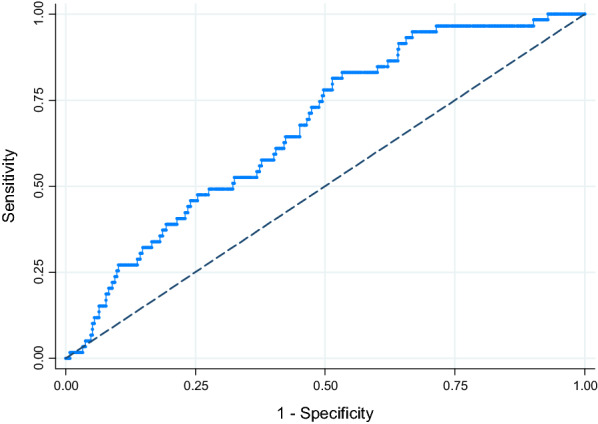
Receiver operating characteristic curve for interpersonal violence over a 3-year period in Dutch patients with schizophrenia spectrum disorders (*N* = 637).

**Figure 2:**
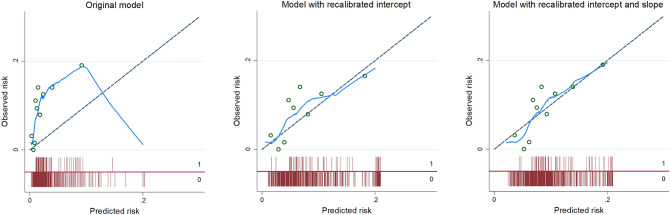
Calibration plots for the original and recalibrated models.

Discrimination, as measured by the AUC, was moderate at 0.67 (95% CI 0.61–0.73). The ROC curve is shown in Fig. 1. The original model had low sensitivity (25%) and high specificity (90%) at the default threshold of 5%. The same pattern was observed for the PPV (21%) and NPV (92%) (Table 2). Calibration-in-the-large was satisfactory, with a ratio between predicted and observed events of 1.2 and a Brier score of 0.09 (Table 3). At the individual level, however, risks were systematically underestimated. This was remedied by recalibration of the intercept and slope (updating step ii) (Fig. 2 and, for the model formula, Table S6). Re-estimation of coefficients (updating step iii) was therefore not necessary. When using a threshold of 10%, the model with the recalibrated intercept and slope also offered a better balance between sensitivity (47%) and specificity (73%) than the original model (Table 2).

**Table 2:** Discrimination metrics for the original and recalibrated models.

Threshold	Sensitivity	Specificity	PPV	NPV
**Original model**				
5	25 (16–38)	90 (87–92)	21 (13–31)	92 (90–94)
10	2 (0–9)	97 (96–98)	6 (1–27)	91 (88–93)
15	0 (0–6)	99 (98–100)	0*	91 (88–93)
20	0 (0–6)	100 (100–100)	0*	91 (88–93)
**Model with recalibrated intercept**				
5	83 (72–91)	42 (38–46)	13 (10–16)	96 (93–98)
10	46 (34–58)	75 (72–79)	16 (11–22)	93 (90–95)
15	29 (19–41)	86 (83–88)	17 (11–26)	92 (90–94)
20	27 (17–40)	90 (87–92)	21 (13–31)	92 (90–94)
**Model with recalibrated intercept and slope**				
5	97 (88–99)	14 (12–17)	10 (8–13)	98 (92–99)
10	47 (35–60)	73 (70–77)	15 (11–21)	93 (90–95)
15	27 (17–40)	89 (86–91)	20 (12–29)	92 (90–94)
20	7 (3–16)	95 (93–96)	12 (5–27)	91 (88–93)

Data are given as percentages, with 95% confidence intervals between parentheses.

*PPV* positive predictive value, *NPV* negative predictive value.

*The number of predicted events was too low (*k* < 5) to reliably calculate a confidence interval.

**Table 3:** Calibration-in-the-large of the original and recalibrated models.

Threshold	PR	PR:OB ratio	Brier score
**Original model**			
5%	73	1.2	0.09
10%	17	0.3	
15%	4	< 0.1	
20%	2	< 0.1	
**Model with recalibrated intercept**			
5%	387	6.6	0.08
10%	170	2.9	
15%	100	1.7	
20%	76	1.3	
**Model with recalibrated intercept and slope**			
5%	553	9.4	0.08
10%	182	3.1	
15%	82	1.4	
20%	34	0.6	

*PR* predicted number of events, *OB* observed number of events.

## Discussion

In a Dutch sample of 637 general psychiatric patients with schizophrenia spectrum disorders, we evaluated the performance of a newly developed risk assessment tool (OxMIV) in predicting interpersonal violence over 3 years. We found OxMIV performed moderately well, especially considering that it is designed to predict a different outcome (i.e., violent offending within 1 year). The broader definition of the outcome and longer follow-up period may partly explain why we obtained a lower AUC (0.67, 95% CI 0.61–0.73) than previous validation studies of OxMIV^7,8^. At the same time, it is comparable to AUCs reported by validation studies of other more resource-intensive tools^4,^, and the current study is an external validation using a different clinically informative outcome. Furthermore, unlike other tools, where calibration has not been reported, OxMIV demonstrated good calibration in the large. In addition, we showed that the performance of OxMIV can be optimised with model updating: calibration at the individual level was adequate after recalibration of the intercept and slope. This is important methodologically as it provides an approach to test the performance of prediction models and risk assessment tools for a different outcome than in their derivation/development studies. 

Strengths of this study include the representativeness of the sample, use of multiple data sources for the outcome, prespecification of the methods, and presentation of a wide range of performance measures. However, there are some limitations. First, most predictors were defined differently than in the derivation study (Table S1). The distribution of predictors differed as well. Of note, the proportion of men was larger (77% vs. 49%), mean age lower (27 years vs. 44 years) and recent treatment with antipsychotics more common (95% vs. 54%) in the current sample (Table 1) than in the derivation sample (Table S7). These differences may have hampered the performance of OxMIV. At the same time, they reflect the profile of patients presenting at mental health services in the Netherlands where information is not always available to align predictors exactly with those in the derivation study, and external validations with patient groups with different baseline characteristics provide evidence whether a tool's performance can be maintained in real-world clinical settings and practice. Another limitation was the relatively small number of patients with the outcome. It has been suggested that ≥ 100 events are required to reliably measure predictive accuracy^21^. For this reason, the findings may be considered preliminary rather than definitive. Finally, missing data may have introduced bias. However, multiple imputation would have reduced this bias in the predictors^22^, and patients with and without outcome data were similar on nearly all predictors.

The findings suggest that OxMIV is suitable for predicting violent behaviour in Dutch patients with schizophrenia spectrum disorders. Clinicians are advised to use the probability scores generated by the model with the recalibrated intercept and slope, as it had the best individual-level calibration and discrimination was worse at the chosen thresholds. This revised model can be accessed on the OxRisk website (https://oxrisk.com). The original model can be used to screen patients for low risk of violence (< 5%), as facilitated by the high NPV (92%). The low PPV (21%) suggests that patients should be assessed further if classified as high risk (≥ 5%). There remains a need for validation studies in which variable definitions more closely match those in the derivation study and the number of events is higher. Comparing the performance of OxMIV against other tools or investigating its clinical feasibility could also be considered.

## Supplementary Information

Supplementary Information.

## Data Availability

Supporting data for this study are not available, as the participants did not agree for these to be shared publicly.
