# Supplementary material for: Validation and recalibration of OxMIV in predicting violent behaviour in patients with schizophrenia spectrum disorders
Source: Sci Rep. 2022 Jan 10;12:461. doi: 10.1038/s41598-021-04266-9 (PMC8748785; doi:10.1038/s41598-021-04266-9)
Supplement: Supplementary file 1 — Supplementary Information. [file 41598_2021_4266_MOESM1_ESM.docx]

SUPPLEMENTARY MATERIAL

**List of Genetic Risk and Outcome of Psychosis (GROUP) investigators**

Therese van Amelsvoort^1^, Agna Bartels-Velthuis^2^, Richard Bruggeman^2,3^, Wiepke Cahn^4^, Lieuwe de Haan^5,6^, René Kahn^4,7^, Frederike Schirmbeck^5,6^, Claudia Simons^1,8^, Jim van Os^4,9^

^1^Department of Psychiatry and Neuropsychology, Maastricht University Medical Centre, Maastricht, the Netherlands. ^2^University Centre for Psychiatry, University Medical Centre Groningen, Groningen, the Netherlands. ^3^Department of Clinical and Developmental Neuropsychology, University of Groningen, Groningen, the Netherlands. ^4^Department of Psychiatry, University Medical Centre Utrecht, Utrecht, the Netherlands. ^5^Department of Psychiatry, Academic Medical Centre, Amsterdam, the Netherlands. ^6^Arkin Institute for Mental Health, Amsterdam, the Netherlands. ^7^Department of Psychiatry, Icahn School of Medicine at Mount Sinai, New York, NY, USA. ^8^GGzE Institute for Mental Health Care, Eindhoven, the Netherlands. ^9^Department of Psychosis Studies, King’s College London, London, United Kingdom.

**Table S1.** Definitions of the predictors in the current study, alongside those in the derivation study.

|  | **Definition** | |
| --- | --- | --- |
| **Predictor** | Derivation study | Current study |
| Male sex | Sex assigned at birth (male v. female) | Same as derivation study |
| Age | Age in years | Same as derivation study |
| Previous violence | Having ever been convicted of any of the following offences: homicide, assault, robbery, arson, rape, sexual coercion, child molestation, indecent exposure, sexual harassment, illegal threats, or intimidation | Having ever physically attacked another person |
| Previous drug misuse | Lifetime diagnosis of drug use disorder (ICD-8: 304; ICD-9: 292, 304, 305 excl. 305A; ICD-10: F11–19) | Lifetime diagnosis of substance abuse (DSM-IV-TR: 305.30–305.90) or dependence (DSM-IV-TR: 304.00–304.90 excl. 304.30) |
| Previous alcohol misuse | Lifetime diagnosis of alcohol use disorder (ICD-8: 291, 303; ICD-9: 291, 303, 305A; ICD-10: F10) | Regular intake of >50 units per week for men and >35 units per week for women, in line with the UK’s NICE guidelines |
| Previous self-harm | Lifetime history of injury of undetermined intent (ICD-8: E980–989; ICD-9: E980–989; ICD-10: Y10–34) | Lifetime history of attempted suicide |
| Educational level | Number of years in education, divided into three categories: (i) lower secondary (<16); (ii) upper secondary (16–18); and (iii) postsecondary (>18) | Highest level of educational attainment, divided into three categories: (i) lower secondary (up to pre-vocational education); (ii) upper secondary (selective secondary education); and (iii) postsecondary (vocational education and higher education) |
| Parental drug or alcohol misuse | Same as for patients | Same as for patients |
| Parental violence | Same as for patients | Expression of anger that is grossly inappropriate or disproportionate to the situation at hand, ranging in seriousness from verbal threats to homicide |
| Sibling violence | Same as for patients | Same as for parents |
| Currently an inpatient | Current episode of care (inpatient v. outpatient) | Unavailable |
| Recent antipsychotic treatment | Use of antipsychotics (ATC: N05A excl. lithium) in the previous six months | Use of antipsychotics in the previous six months |
| Recent antidepressant treatment | Use of antidepressants (ATC: N06A) in the previous six months | Use of antidepressants in the previous six months |
| Recent dependence treatment | Use of medication for addictive disorders (ATC: N07BB, N07BC) in the previous six months | Unavailable |
| Personal income | Disposable personal income, split into deciles of the distribution in the Swedish population | Net monthly income, dived into two categories: (i) unstable (less than minimum wage); and (ii) stable (minimum wage or more) |
| Benefit recipient | Receiving welfare or disability benefits | Not being in paid employment |

ICD, International Statistical Classification of Diseases and Related Health Problems; DSM, Diagnostic and Statistical Manual of Mental Disorders; NICE, National Institute for Health and Care Excellence; ATC, Anatomical Therapeutic Chemical Classification System

**Table S2.** Instruments used to measure the predictors.

| **Instrument** | **Method of data collection** | **Predictor** |
| --- | --- | --- |
| Intake form | Structured interview | Male sex |
|  |  | Age |
|  |  | Educational level |
|  |  | Benefit recipient |
| LCS | Semi-structured interview and case note review | Previous violence |
|  |  | Previous self-harm |
|  |  | Recent antipsychotic treatment |
|  |  | Recent antidepressant treatment |
| CIDI-SAM | Structured interview | Previous drug misuse |
|  |  | Previous alcohol misuse |
|  |  | Parental drug or alcohol misuse |
| CASH | Semi-structured interview | Parental violence |
|  |  | Sibling violence |
|  |  | Unstable income |

LCS, Life Chart Schedule; CIDI-SAM, Substance Abuse Module of the Composite International Diagnostic Interview; CASH, Comprehensive Assessment of Symptoms and History

**Table S3.** Patients with missing data (*N* = 637).

| **Predictor** | ***n* (%)** |
| --- | --- |
| Male sex | 0 (0) |
| Age | 0 (0) |
| Previous violence | 84 (13) |
| Previous drug misuse | 52 (8) |
| Previous alcohol misuse | 13 (2) |
| Previous self-harm | 27 (4) |
| Educational level | 6 (1) |
| Parental drug or alcohol misuse | 294 (46) |
| Parental violence | 359 (56) |
| Sibling violence | 249 (39) |
| Recent antipsychotic treatment | 44 (7) |
| Recent antidepressant treatment | 0 (0) |
| Unstable income | 177 (28) |
| Benefit recipient | 37 (6) |

**Table S4.** Correlations between missingness in each predictor and values of the others (*N* = 1013).

|  | **Missingness** | | | | | | | | | | |
| --- | --- | --- | --- | --- | --- | --- | --- | --- | --- | --- | --- |
| **Values** | 1 | 2 | 3 | 4 | 5 | 6 | 7 | 8 | 9 | 10 | 11 |
| Male sex† | .05 | .06 | <\|.01\| | .04 | .03 | <\|.01\| | -.02 | .03 | .06 | -.03 | .02 |
| Age† | -.09** | -.05 | -.04 | -.03 | -.05 | .23*** | .21*** | -.04 | -.06 | -.08** | -.02 |
| Previous violence (1) | - | .04 | -.03 | -.05 | -.03 | <\|.01\| | -.04 | .01 | .04 | -.04 | .01 |
| Previous drug misuse (2) | -.02 | - | -.04 | -.04 | <\|.01\| | <\|.01\| | .01 | <\|.01\| | -.04 | -.04 | -.06 |
| Previous alcohol misuse (3) | -.02 | -.02 | - | .05 | <\|.01\| | .08** | .03 | -.02 | .04 | -.04 | .02 |
| Previous self-harm (4) | -.06 | <\|.01\| | <\|.01\| | - | -.04 | -.04 | .03 | -.05 | -.04 | .01 | .02 |
| Educational level (5) | -.01 | -.02 | -.04 | <\|.01\| | - | -.02 | <\|.01\| | -.05 | -.03 | .01 | .01 |
| Parental drug or alcohol misuse (6) | -.08 | .04 | -.04 | <\|.01\| | .05 | - | -.03 | -.05 | .04 | <\|.01\| | .01 |
| Parental violence (7) | -.08 | -.03 | -.03 | .02 | † | -.02 | - | <\|.01\| | -.05 | .02 | .06 |
| Sibling violence (8) | -.01 | -.03 | -.01 | -.01 | -.02 | .08 | .06 | - | <\|.01\| | .05 | -.02 |
| Recent antipsychotic treatment (9) | -.02 | -.01 | <\|.01\| | -.01 | .01 | -.05 | -.01 | .07* | - | .04 | .04 |
| Recent antidepressant treatment† | -.09** | -.05 | -.03 | -.07* | <\|.01\| | -.05 | -.04 | -.04 | -.13*** | -.02 | -.10** |
| Unstable income (10) | .04 | -.01 | <\|.01\| | .08* | -.02 | .09* | .09* | -.05 | .01 | - | .09* |
| Benefit recipient (11) | .04 | -.02 | .01 | .04 | <\|.01\| | -.06 | -.02 | .06 | .03 | .07* | - |

* *p* <.05

** *p* <.01

*** *p* <.001

† No missing data

**Table S5.** Comparisons between patients with (*n* = 637) and without (*n* = 376) outcome data.

|  | **Outcome data** | | **Comparison** | |
| --- | --- | --- | --- | --- |
| **Predictor** | Yes | No | Test statistic (*df*) | *p* |
| Male sex | 493 (77%) | 297 (79%) | χ^2^ (1) = 0.35 | .554 |
| Age | M (SD) = 27 (7) | M (SD) = 27 (7) | *t* (1011) = 1.09 | .277 |
| Previous violence | 115 (21%) | 68 (20%) | χ^2^ (1) = 0.06 | .808 |
| Previous drug misuse | 118 (20%) | 85 (25%) | χ^2^ (1) = 2.61 | .106 |
| Previous alcohol misuse | 79 (13%) | 35 (10%) | χ^2^ (1) = 1.92 | .165 |
| Previous self-harm | 142 (23%) | 81 (23%) | χ^2^ (1) = 0.04 | .834 |
| Educational level  Lower secondary  Upper secondary  Postsecondary | 265 (42%)  177 (28%)  189 (30%) | 194 (52%)  73 (20%)  105 (28%) | χ^2^ (2) = 12.18 | .002 |
| Parental drug or alcohol misuse | 63 (18%) | 33 (19%) | χ^2^ (1) = 0.08 | .775 |
| Parental violence | 13 (5%) | 9 (6%) | χ^2^ (1) = 0.60 | .439 |
| Sibling violence | 47 (12%) | 28 (14%) | χ^2^ (1) = 0.29 | .590 |
| Recent antipsychotic treatment | 565 (95%) | 330 (96%) | χ^2^ (1) = 0.07 | .792 |
| Recent antidepressant treatment | 147 (23%) | 74 (20%) | χ^2^ (1) = 1.60 | .206 |
| Unstable income | 369 (80%) | 225 (82%) | χ^2^ (1) = 0.54 | .463 |
| Benefit recipient | 276 (46%) | 192 (54%) | χ^2^ (1) = 5.42 | .020 |

**Table S6.** Model formulas.

| **Model** | **Formula** |
| --- | --- |
| Original model | logit(*p*) = a + Σ(b_i_*X_i_) |
| Model with recalibrated intercept | logit(*p*) = -2.1374 + Σ(b_i_*X_i_) |
| Model with recalibrated intercept and slope | logit(*p*) = -2.1150 + 0.5666*Σ(b_i_*X_i_) |

**Table S7.** Summary statistics for the predictors in the derivation sample (*N* = 58 771).

| **Predictor** | **Summary** |
| --- | --- |
| Male sex | 29 077 (49%) |
| Age | M (SD) = 44 (13) |
| Previous violence | 9212 (16%) |
| Previous drug misuse | 7123 (12%) |
| Previous alcohol misuse | 8897 (15%) |
| Previous self-harm | 11 510 (20%) |
| Educational level  Lower secondary  Upper secondary  Postsecondary | 17 814 (35%)  26 449 (52%)  6489 (13%) |
| Parental drug or alcohol misuse | 5214 (11%) |
| Parental violence | 3203 (7%) |
| Sibling violence | 4028 (7%) |
| Currently an inpatient | 18 160 (31%) |
| Recent antipsychotic treatment | 18 401 (54%) |
| Recent antidepressant treatment | 13 255 (39%) |
| Recent dependence treatment | 1030 (3%) |
| Personal income  First decile (lowest)  Fifth decile  Tenth decile | 5444 (9%)  9169 (16%)  2009 (3%) |
| Benefit recipient | 37 210 (64%) |

Protocol for “Predicting violence in patients with schizophrenia spectrum disorders: a Dutch validation study of OxMIV”

**OxMIV**

Oxford Mental Illness and Violence (OxMIV) is a freely available online tool for predicting violence in people with severe mental illness (https://oxrisk.com/oxmiv). It is one of the tools that make up OxRisk, a project by the Forensic Psychiatry and Psychology Group at the University of Oxford.

OxMIV is based on a logistic regression model with 16 predictors, covering sociodemographic, criminal and clinical characteristics. The model was derived using a sample of nearly 60 000 Swedish individuals with schizophrenia spectrum or bipolar disorder^1^.

Upon entry of the 16 items, OxMIV estimates the probability of violent offending within 12 months. This estimate is expressed as a percentage, capped at 20%. A classification of ‘low risk’ (<5%) or ‘increased risk’ (≥5%) is also given. Some items may be set to ‘unknown’. If this is the case, OxMIV returns a range of risk levels. The lower and upper bounds of a range correspond to the minimum and maximum risk levels that would have been possible to obtain with complete information.

**Objective**

The objective of the proposed study is to evaluate the performance of OxMIV in a sample of patients with schizophrenia spectrum disorders in the Netherlands (model validation). If necessary, we will also attempt to adjust OxMIV for use in this population (model updating).

**Study setting and participants**

Data were collected as part of a larger research project, called Genetic Risk and Outcome in Psychosis (GROUP)^2^. The GROUP project was conducted by four university hospitals and affiliated mental health centres in the Netherlands.

Throughout 2004, consecutive patients were invited to participate if they met the following criteria: (i) age between 16 and 50; (ii) good command of the Dutch language; (iii) Diagnostic and Statistical Manual of Mental Disorders, Fourth Edition, Text Revision (DSM-IV-TR) diagnosis of schizophrenia or other non-affective psychotic disorder; and (iv) able and willing to give written informed consent. Patients were followed up after three years.

**Outcome**

The outcome will be physical abuse of another person during the three years after baseline, ascertained from clinical case notes and patient interviews.

**Preliminary data on sample size and event rates**

Preliminary data suggest a total sample size of 637, with an event rate of 9% (*n* = 59). By comparison, the event rate in the derivation sample was 1%. Likely explanations for the higher event rate in the Dutch sample are the use of different data sources (clinical case notes and patient interviews v. criminal records) and the longer follow-up period (three years v. one year).

Since the objective of the study is model validation and possibly updating, no formal sample size calculation will be performed.

**Selection of predictors**

In instances where two or more variables are available for the same item, we will use the strategy described in Table 1 to choose one. Next steps will be taken only if the previous step does not help doing so.

**Table 1.** Strategy for selecting predictors.

| **Step** | **Description** |
| --- | --- |
| i | The variable whose definition is closest to its equivalent in the derivation study. |
| ii | The variable whose prevalence in the Dutch sample is closest to that of its equivalent in the derivation sample. |
| iii | The variable whose effect on the outcome is closest to that of its equivalent in the derivation study. |

**Completely and partly missing predictors**

If a predictor is completely missing, we will try using a proxy first. If a suitable proxy is unavailable, we will assign the derivation sample mean to all participants^3^. Multiple imputation will be used for partly missing predictors^4^.

**Statistical analysis**

We will first assess the performance of the original model. If performance is poor, we will also explore the feasibility of adapting the model to the patient population in the Netherlands. For this, we will consider a series of statistical updating steps suggested previously^5,6^ (Table 2). Next steps will be taken only if the model still performs poorly after the previous step.

**Table 2.** Strategy for model updating.

| **Step** | **Description** |
| --- | --- |
| i | Recalibrate the intercept. This adjusts for systematic differences in baseline risk not explained by the predictors. |
| ii | Recalibrate the intercept and slope. Apart from rescaling, the effect of each predictor on the outcome is assumed to be the same as in the derivation study. |
| iii | Re-estimate one or more coefficients. We will re-estimate as few coefficients as possible, because precision is expected to be lower than in the derivation study and the objective of the proposed study is model validation (not model development). |

Performance will be assessed in terms of calibration and discrimination. Calibration refers to the agreement between observed and expected outcomes. We will assess calibration both ‘in the large’ (i.e., whether the model adequately estimates the event rate across the whole sample) and at individual level. Discrimination is the ability of the model to distinguish individuals with the outcome from those without the outcome.

In assessing calibration and discrimination, we will use graphs and summary statistics as appropriate. These will include calibration plots, the *c*-index, and sensitivity and specificity^7,8^.

**Date: May 15^th^, 2020**

**References**

1. Fazel, S. *et al.* Identification of low risk of violent crime in severe mental illness with a clinical prediction tool (Oxford Mental Illness and Violence tool [OxMIV]): a derivation and validation study. *Lancet Psychiatry* **4,** 461–468 (2017).
2. Korver, N. *et al.* Genetic Risk and Outcome of Psychosis (GROUP), a multi-site longitudinal cohort study focused on gene-environment interaction: objectives, sample characteristics, recruitment and assessment methods. *Int. J. Methods Psychiatr. Res.* **21,** 205–221 (2012).
3. Janssen, K. J. *et al.* Dealing with missing predictor values when applying clinical prediction models. *Clin. Chem.* **55,** 994–1001 (2009).
4. Held, U. *et al.* Methods for handling missing variables in risk prediction models. *Am. J. Epidemiol.* **184,** 545–551 (2016).
5. Steyerberg, E. W. *Clinical Prediction Models: a Practical Approach to Development, Validation, and Updating* (Springer, 2009).
6. Su, T.-L., Jaki, T., Hickey, G. L., Buchan, I. & Sperrin, M. A review of statistical updating methods for clinical prediction models. *Stat. Methods Med. Res.* **27,** 185–197 (2018).
7. Steyerberg, E. W. *et al.* Assessing the performance of prediction models: a framework for traditional and novel measures. *Epidemiology* **21,** 128–38 (2010).
8. Collins, G. S. *et al.* External validation of multivariable prediction models: a systematic review of methodological conduct and reporting. *BMC Med. Res. Methodol.* **14,** 40 (2014).
